# Faith, Cancer, and Compromise: Managing Acute Myeloid Leukemia and Metastatic Triple-Negative Breast Cancer in a Jehovah's Witness Patient

**DOI:** 10.7759/cureus.93184

**Published:** 2025-09-25

**Authors:** Andy Li, Parima Saxena, Aye M Thida, Armine Bagdasaryan, Mary Hanna, Jordonna Brown

**Affiliations:** 1 Internal Medicine, State University of New York Downstate Health Sciences University, Brooklyn, USA; 2 Hematology and Medical Oncology, State University of New York Downstate Health Sciences University, Brooklyn, USA; 3 Pathology, State University of New York Downstate Health Sciences University, Brooklyn, USA

**Keywords:** acute myeloid leukemia (aml), jehovah's witness, supportive and palliative care, transfusion practices, triple-negative breast cancer

## Abstract

The treatment of acute myelogenous leukemia (AML) is accompanied by several potentially life-threatening adverse effects, the most prevalent of which are cytopenias (anemia, thrombocytopenia, and neutropenia). As such, therapy selection is extremely challenging in patients who cannot tolerate blood transfusions or are unable due to religious convictions, such as those who practice as Jehovah's Witnesses. Additionally, the existence of another primary aggressive neoplasm further narrows the scope of therapeutic options. Here, we present the first reported case of an elderly 72-year-old woman who practices as a Jehovah's Witness, concurrently diagnosed with AML and metastatic triple-negative breast cancer. We reviewed her clinical course, management, and ultimate decision to forgo treatment due to religious beliefs. Treatment of AML in the elderly, notwithstanding transfusion status, is an unmet growing need. Here, we discuss the ethical considerations of cytopenia management in patients undergoing AML treatment and delve into the literature surrounding supportive care in such patients.

## Introduction

Acute myelogenous leukemia (AML) is a lethal cancer that accounts for one-third of all leukemias in adults, with a slightly higher prevalence in men [1,2]. Despite treatment, there is a five-year survival rate of 33%, indicating a poor prognosis for adults receiving the diagnosis [3]. There is an even lower survival rate for adults > 65 years of age, where only 2.1% of patients survived more than 12 months [4,5]. Treatment options are further limited in patients who cannot accept blood transfusions due to the high risk of cytopenias associated with therapy. There is no current standard of care to help guide treatment in such situations, as transfusion support is a crux for managing the toxic side effects of chemotherapy.

Treatment options become even more limited when AML occurs concurrently with another primary malignancy. The management of multiple primaries that do not share a common therapy is a poor prognostic factor, especially when each of the primary malignancies is considered highly aggressive. The combination of multiple primaries in conjunction with an inability to receive transfusion support creates a complex question regarding treatment risks and benefits.

Here, we present the ethical considerations of treatment in the first reported case of an elderly Jehovah's Witness patient with a simultaneous diagnosis of AML and metastatic triple-negative breast cancer. We also review the literature of previously treated Jehovah's Witness patients with AML who did not accept blood transfusions to provide guidance for therapy options in a clinically and ethically challenging scenario.

## Case presentation

**Figure 1:**
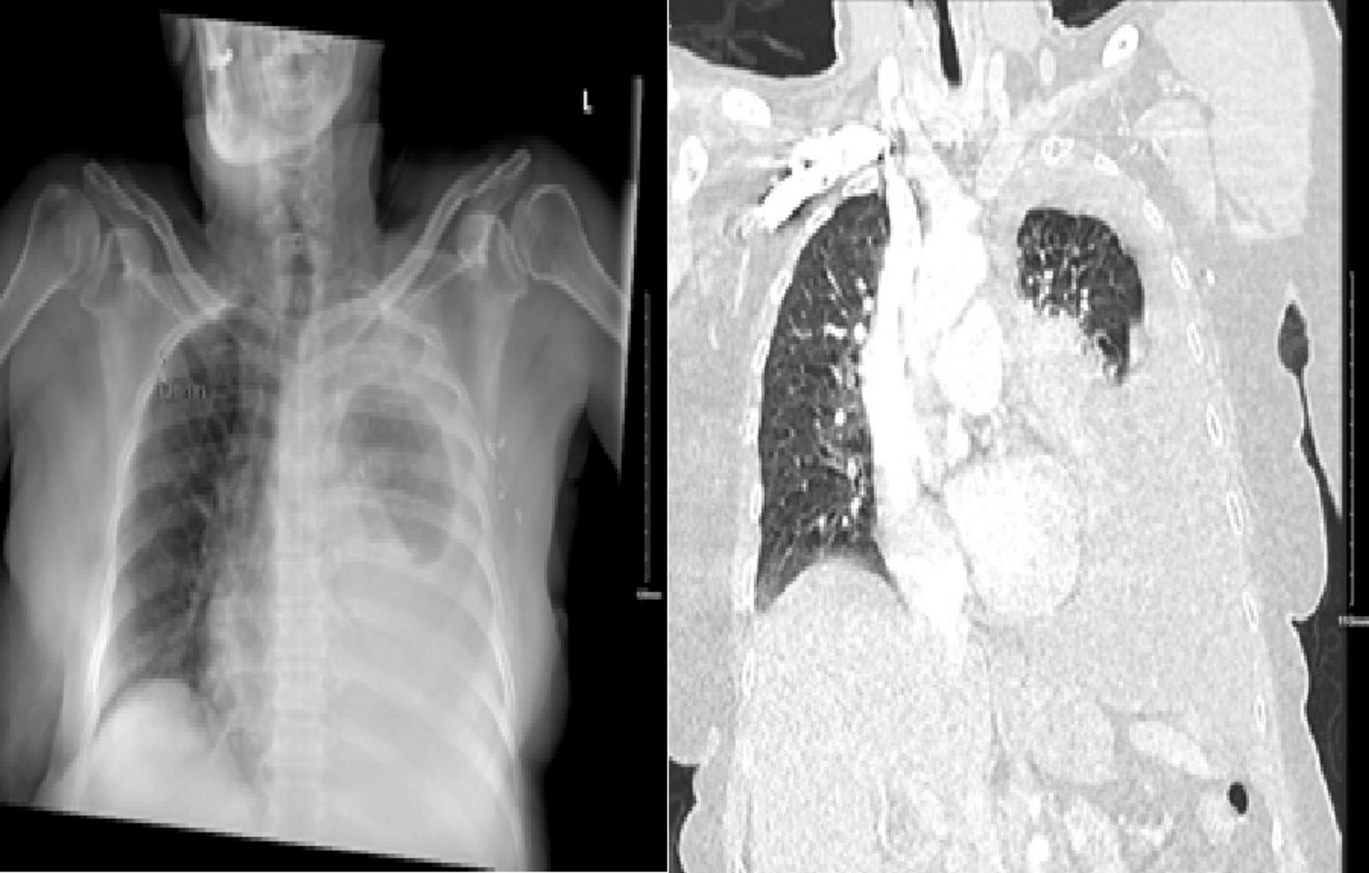
Chest X-ray and chest CT (lung view) demonstrating a large left-sided pleural effusion

A 72-year-old Jehovah's Witness woman with a past medical history of hypertension, hyperlipidemia, and hormone receptor (HR)-negative/human epidermal growth factor receptor 2 (HER2)-positive, stage IA (pT1cpN1M0) left breast cancer, treated with radical mastectomy, adjuvant docetaxel, carboplatin, trastuzumab (TCH), and adjuvant radiation and having completed maintenance trastuzumab about three years ago, now on active surveillance, initially presented to the emergency department for shortness of breath for the past two weeks. Vitals on the initial examination were significant for an at-rest O2 saturation of 94% that decreased to 90% on exertion and a heart rate of 104 beats per minute. Physical examination was significant for decreased breath sounds over the left lung fields. A chest X-ray showed a large left pleural effusion, further confirmed by a chest CT (Figure 1). A thoracentesis was performed, and the collected fluid was sent for further analysis. Given the patient's marked improvement in symptoms, she was discharged from the hospital and planned to follow up on the results with the pulmonology team.

She returned to the emergency department 10 days later for the re-emergence of her shortness of breath, now accompanied by chest pain. Vitals on this admission were only significant for a heart rate of 99 beats per minute. On this admission, the patient's automated complete blood count showed a hemoglobin of 11.8 g/dL (reference range: 12-16 g/dL) (previous admission was 13.7 g/dL), a blast count of 16.5%, a monocyte count of 33.5%, and 5 nucleated red blood cells per 100 white blood cells (Table 1).

**Table 1:** Laboratory parameters of the patient in both hospitalizations WBC: white blood cell, RBC: red blood cell

Parameters	Final available laboratory results	Second admission	Initial admission	Reference range
WBC total	91.70 K/uL	6.13 K/uL	3.48 K/uL	4.50-10.90 K/uL
Blast count (peripheral blood)	Not reported	16.5%	Not reported	0% (normally absent)
Monocyte count	Not reported	33.5%	Not reported	Not reported
Hemoglobin	8.1 g/dL	11.8 g/dL	13.7 g/dL	12-16 g/dL
Nucleated RBCs (per 100 WBCs)	Not reported	5/100 WBCs	Not reported	0/100 WBCs (normally absent)
Platelets	35 K/uL	150 K/uL	227 K/uL	130-400 K/uL

**Figure 2:**
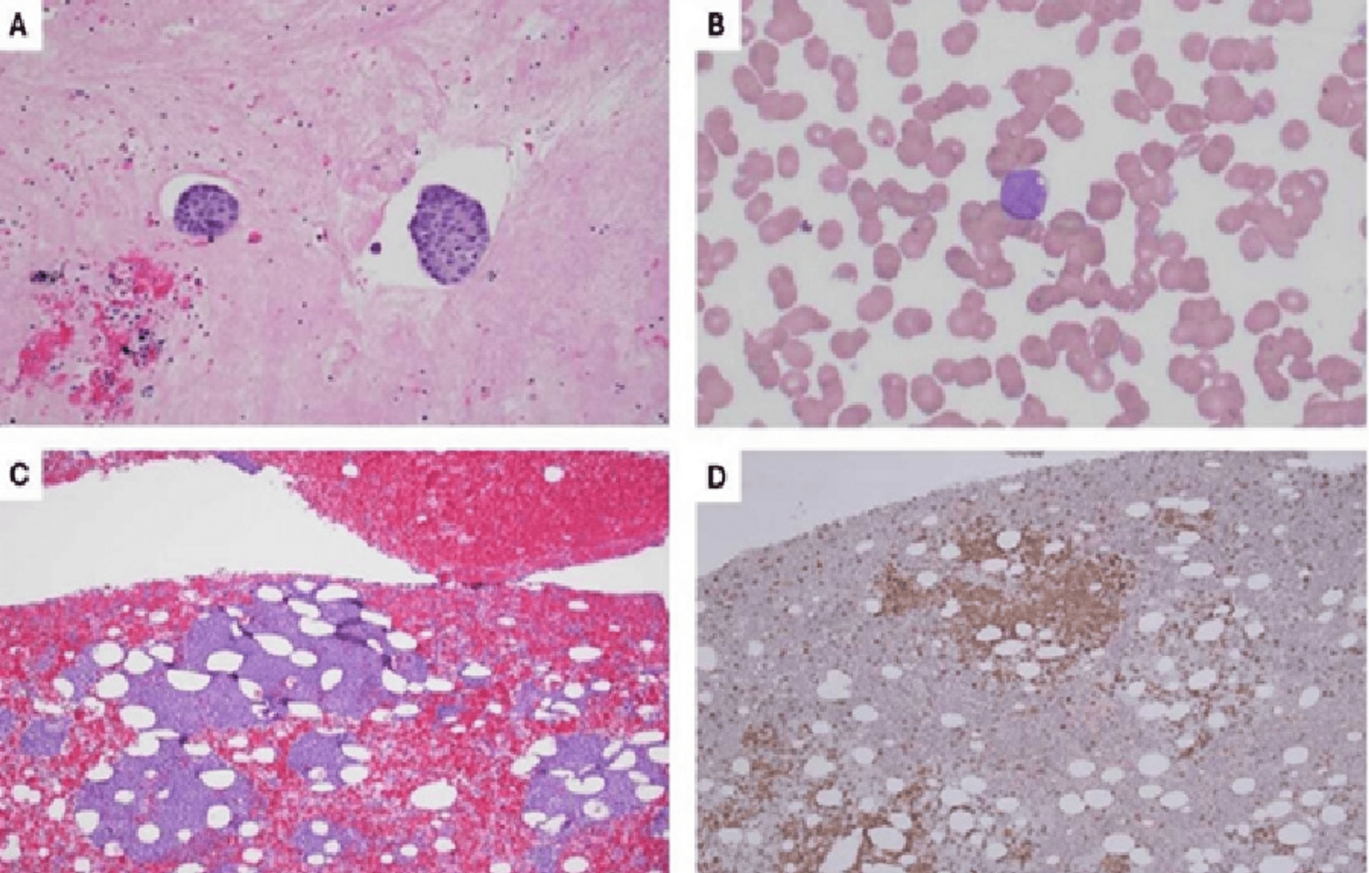
Pleural fluid with metastatic carcinoma and acute myeloid leukemia diagnosed later Pleural fluid revealed clusters of neoplastic epithelial cells (A) (H&E, 200× magnification) positive for MOC31, BER-EP4, and GATA-3 (weak) (data not shown), supporting metastatic carcinoma of breast origin. Circulating blasts (17%) were noted in the peripheral blood (B, one representative blast) (Wright-Giemsa stain, 400× magnification), and bone marrow showed myeloblasts (C, marrow clot) (H&E, 100× magnification), 50%-60%, positive for CD117 (D, marrow clot) (immunohistochemistry, 100× magnification), CD34 (dim), myeloperoxidase, CD13, CD33, and HLA-DR (data not shown). H&E: hematoxylin and eosin

Additionally, the pleural fluid analysis from prior thoracentesis disclosed the presence of scattered clusters of atypical epithelial cells, staining positive for epithelial-specific antigen (MOC31), anti-human epithelial antigen (BER-EP4), and GATA binding protein 3 (weakly), and negative for wingless-type integration family member 1 (WNT1) and calretinin, consistent with a diagnosis of metastatic breast carcinoma (Figure 2A). The cancer cells did not express HR/HER2, indicating that it was a triple-negative, metastatic breast cancer.

**Figure 3:**
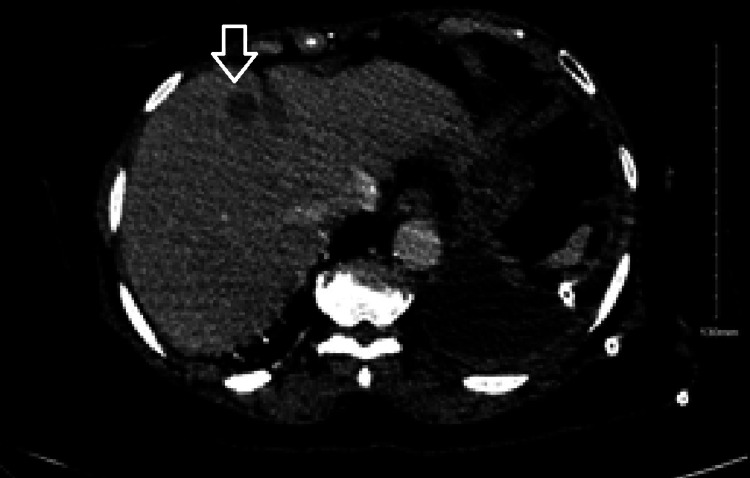
Abdominal CT demonstrating metastatic liver lesions (arrow), as seen by a hypodense lesion noted on the liver

Repeat chest imaging confirmed the recurrence of the large left pleural effusion present on previous admission, and a pigtail catheter was placed. Imaging of the abdomen and pelvis revealed two soft tissue lesions in the right hepatic lobe and two additional lesions in section 8 of the liver (Figure 3), likely metastatic disease from the breast. Imaging of the brain did not show lesions. The patient was admitted for the management of clinical manifestations of metastatic breast cancer and additional investigation into the presence of elevated blast cell counts in the peripheral blood (Figure 2B). A bone marrow biopsy unveiled 25% myeloblasts in the marrow aspirate (Figure 2C, 2D), with flow cytometry showing FMS-like tyrosine kinase 3 (FLT3) and nucleophosmin (NPM1) mutations, confirming the diagnosis of a second primary malignancy, acute myelogenous leukemia (AML). There was no evidence to suggest treatment-related AML.

The patient's beliefs about blood transfusion support were clarified: she did not accept whole or partial components of human blood. Treatment of either the AML or the breast cancer would likely result in cytopenias without the possibility of transfusion. Furthermore, there were no shared therapies between the two cancers. It was determined that attempting to treat either cancer would likely hasten the patient's demise, and goals of care discussion were initiated with the patient. The patient accepted that there are no safe treatment options, and she agreed to begin the transition toward receiving hospice care with comfort measures. She shortly passed away within a few weeks.

## Discussion

Here, we summarize a case of two primary neoplasms with a complicated consideration of ethical principles and patient values in management. Our case highlights the importance of considering patients' beliefs in joint decision-making and cancer-directed therapies. When discussing treatment options with our patient, multidisciplinary team management and an extensive literature review were critical to aid in decision-making.

Reports of treatment for AML in Jehovah's Witness patients are sparse in the literature, particularly in elderly patients (age > 65). One report discusses management in a 61-year-old woman, being the highest age group discussed from our literature search [6].

The treatment of AML revolves around the 7+3 backbone induction regimen prior to autologous stem cell transplant, consisting of cytarabine for seven days along with daunorubicin/idarubicin or mitoxantrone (for patients over 60 years of age) for three days [7]. In older patients, typically over 70 years of age, induction with azacitidine and venetoclax is the most commonly used regimen [8]. We also considered the use of gemtuzumab + ozogamicin, especially since our patient expressed CD33, and as per the ALFA-701 study [9]. However, the adverse effect of cytopenias cannot be discounted, and the highest risk of developing myelosuppression is upon initiation of the therapy [9]. Since our patient expressed the FLT3 mutation, we also considered the role of midostaurin or quizartinib [7,10]. However, since our patient adheres to her beliefs as a member of Jehovah's Witnesses, this complicates the consideration of therapy options due to the high risk of cytopenias seen in many of these regimens. There are currently no established guidelines for the treatment of AML in patients who cannot receive transfusion support [7].

We considered initiating azacitidine monotherapy for our patient, as it tends to be less toxic than conventional chemotherapy at the cost of being less likely to achieve complete remission [11]. There has been one reported Jehovah's Witness patient who had been treated with azacitidine alone and had achieved complete remission [11]. However, this particular case involved a young man without other comorbidities, which is a stark contrast to our patient. Due to its limited proven success at achieving remission in the younger population, let alone those in the elderly group, we evaluated its possible benefit to at least prolonging our patient's life and believed starting azacitidine would cause harm to the patient. Furthermore, our patient also presented with another highly aggressive neoplasm: triple-negative breast cancer with confirmed metastasis to both the lungs and the liver.

We summarize a review of case reports describing the treatment of AML in Jehovah's Witness patients in Table 2.

**Table 2:** Summary of case reports in the literature discussing the management of AML in Jehovah's Witness patients *5/9 reduced dose of either etoposide/methotrexate, araC/thioguanine, araC, or gemtuzumab ozogamicin **1/9 lost to follow-up, 1/9 not reported, 0/9 achieved durable remission ***Swapped to araC + mitoxantrone araC: cytarabine arabinoside

Patient age	Cytogenetics	Treatment	Adverse effects	Outcome	Source
Not specified, matched to control	Not reported	4/9 full-dose araC + idarubicin*	4/9 severe anemia, 1/9 uncontrolled bleeding	5/9 early death, 2/9 complete remission, then relapsed**	[3]
33	None	Azacitidine	Abscess, pancytopenia	Complete remission	[11]
33	ASXL1	Venetoclax + azacitidine	Pulmonary embolism, neutropenia	Complete remission	[11]
35	Not performed	Vinblastine***	Pancytopenia, bilateral pneumonia	Deceased	[12]
40	None	Gemtuzumab + ozogamicin	Pancytopenia	Complete remission	[13]
48	SF3B1	Venetoclax + azacitidine	Pancytopenia	Complete remission	[11]
52	None	Peg-asparaginase, vincristine, methylprednisolone	Peripheral neuropathy, thrombosis	Complete remission	[14]
61	None	Gemtuzumab + ozogamicin	Pancytopenia	Complete remission	[6]

Unfortunately, there are no shared treatment options between the two neoplasms, and giving chemotherapy for both cancers at once would likely hasten our patient's death, especially without the use of transfusion support. On the other hand, treatment of just one cancer, such as using azacitidine as a mono-agent for AML, would likely result in cytopenic toxicity, but ignoring her metastatic breast cancer would also lead to unfavorable outcomes. Overall, we deemed the risk of treatment outweighed the benefits. It was agreed upon that transitioning from management of her two primary neoplasms to hospice care with comfort measures would provide her with the highest quality of life.

## Conclusions

Here, we highlight management considerations for a patient with triple-negative breast cancer and AML with ethical considerations for transfusion support. The case highlights the interplay of religion and medicine, and the importance of regarding patients' religious persuasions when they are central to their care. We review the literature regarding treatment decisions for AML in Jehovah's Witness patients and underscore the importance of literature-based guidance in managing such complex cases.
